# The study on the inverse problem of applied current thermoacoustic imaging based on generative adversarial network

**DOI:** 10.1038/s41598-021-02291-2

**Published:** 2021-11-25

**Authors:** Liang Guo, Su Li, Xiangye Wang, Caihong Zeng, Chunyu Liu

**Affiliations:** College of Information and Control Engineering, China University of Petroleum, Qingdao, 266580 Shandong People’s Republic of China

**Keywords:** Imaging, Ultrasound

## Abstract

Applied Current Thermoacoustic Imaging (ACTAI) is a new imaging method which combines electromagnetic excitation with ultrasound imaging, and takes ultrasonic signal as medium and biological tissue conductivity as detection target. Taking the high contrast advantage of Electrical Impedance Tomography (EIT) and high resolution advantage of ultrasound imaging, ACTAI has broad application prospects in the field of biomedical imaging. Although ACTAI has high excitation efficiency and strong detectable Signal-to-Noise Ratio, yet while under low frequency electromagnetic excitation, it is still a big challenge to reconstruct a high-resolution image of target conductivity. This paper proposes a new method for reconstructing conductivity based on Generative Adversarial Network, and it consists of three main steps: firstly, use Wiener filtering deconvolution to restore the electrical signal output by the ultrasonic probe to a real acoustic signal. Then obtain the initial acoustic source image with filtered backprojection technology. Finally, match the conductivity image with the initial sound source image, which are used as training samples for generating the adversarial network to establish a deep learning model for conductivity reconstruction. After theoretical analysis and simulation research, it is found that by introducing machine learning, the new method can dig out the inverse problem solving model contained in the data, which further reconstruct a high-resolution conductivity image and has strong anti-interference characteristics. The new method provides a new way to solve the problem of conductivity reconstruction in Applied Current Thermoacoustic Imaging.

## Introduction

As a new type of functional imaging technology, Electrical Impedance Tomography^1–3^ is capable of nondestructive detection of changes in electrical parameters of biological tissues, and then obtain the physiological and pathological states of the tissues. It has a broad application prospect in the field of biomedical imaging. However, the low resolution of traditional Electrical Impedance Tomography has become a bottleneck restricting its further development. In order to improve the resolution of Electrical Impedance Tomography, the Multi-Physics Coupling Imaging technology based on the combination of electromagnetic field and sound field has become a research hotspot^4–6^, as it can improve the resolution while at the same time retaining the high contrast advantage of Electrical Impedance Tomography. Examples include Microwave Thermoacoustic Imaging^7,8^, Magneto-mediated Thermoacoustic Imaging^9^, and Applied Current Thermoacoustic Imaging^10,11^, etc.

Microwave Thermoacoustic Imaging uses pulsed microwaves to irradiate biological tissues to produce transient thermal expansion, which in turn excites ultrasound signals. However, its penetration depth is limited by the working frequency of the excitation source, so it is difficult to penetrate deep into tissues and organs. In order to increase the penetration depth, Zheng Research Group of Nanyang Technological University in Singapore proposed a Magneto-mediated Thermoacoustic Imaging method. The principle is to apply a MHz-level alternating magnetic field to a conductive target by using the excitation coil to generate an induced electric field inside the target, and then stimulate the Joule heat to obtain thermoelastic ultrasonic signals. The excitation frequency of magneto-mediated thermoacoustic imaging is 20 MHz, and the penetration depth of electromagnetic wave to biological tissues can reach 15 cm. They detected magneto-mediated thermoacoustic signals using high-conductivity metal block samples and obtained thermoacoustic images of the samples. However, for targets with low conductivity, as the detection depth increases, the induced electric field gradually decreases, and the Signal-to-Noise Ratio of the ultrasonic signal continuously decreases, which increases the difficulty of detection. In 2016, Liu research team of the Institute of Electrical Engineering, Chinese Academy of Sciences proposed the Applied Current Thermoacoustic Imaging (ACTAI). Instead of using the above-mentioned induced electric field, they utilize the applied current signal with microsecond pulse width to generate an ultrasonic signal with a stronger Signal to Noise Ratio (SNR). Compared with the induced current, the excitation frequency of the applied current is easier to adjust and it has a higher penetration depth, which improves the energy conversion efficiency and detectable SNR.

As a new type of thermoacoustic imaging technology, ACTAI still has many problems in the high-resolution reconstruction of conductivity, which need to be further solved. In terms of image reconstruction, the current research mainly focuses on the reconstruction algorithm of sound source image, such as time inversion method, filtered back projection^12^ and so on. Although these algorithms can reflect the distribution characteristics of thermoacoustic sources, they cannot obtain the true conductivity distribution. The fundamental reason is that the Signal-to-Noise Ratio of the ultrasound signal is low, while the inverse problem of rebuilding the conductivity image from the ultrasound signal has a strong nonlinearity, which makes the reconstruction process greatly affected by noise, and the reconstruction results of conductivity cannot meet the requirements of functional imaging.

In recent years, inverse problem reconstruction algorithms based on deep learning have been widely studied in the field of medical imaging, such as magnetic resonance super-resolution imaging, low-dose CT imaging, and so on. From the existing research, we find that the deep learning method has such unique advantages as high degree of nonlinearity, fast solving speed, easy access to simulation training dataset, etc. in resolving large-scale nonlinear reconstruction problems. Generative Adversarial Network^13–15^ (GAN) can reconstruct image based on limited data, remove the view artifact^16^ and improve the image SNR^17^, improve the image similarity and get higher image quality. ACTAI, as a complex Multi-Physics Coupling Imaging mode, its inverse conductivity reconstruction problem precisely meets the above characteristics. Therefore, this paper proposes to apply the deep learning network to conductivity reconstruction in ACTAI in order to obtain the real conductivity distribution image.

## Method

The implementation of the new method consists of the following steps: first, the electrical signal measured by the ultrasonic probe is preprocessed by Wiener filter deconvolution^18,19^ to obtain the original acoustic signal emitted by the measured sample. Then the filtered back projection (FBP) is used to reconstruct the sound source image, match the reconstructed sound source image with the conductivity image of the measured sample, and use it as a training sample to train a Generative Adversarial Network. Finally, the trained network is used to process the sound source image to obtain high-resolution conductivity images. This method comprehensively considers the characteristics of the physical model as well as the data-driven model, and constructs a deep neural network specifically for the ACTAI inverse problem, which can effectively improve the imaging resolution and imaging speed.

In ACTAI, the duration of the pulse current is much shorter than the heat conduction time inside the sample, so only the thermoacoustic signal caused by the adiabatic expansion of biological tissues needs to be considered. The thermoacoustic pressure $p{(}{\varvec{r}},t{)}$ generated at field point ${\varvec{r}}$ can be described as below:

1 $${\nabla }^{2}p(r,t)-\frac{1}{{c}_{s}^{2}}\frac{{\partial }^{2}}{\partial {t}^{2}}p(r,t)=-\frac{\beta }{{C}_{P}}\frac{\partial H({r}^{^{\prime}},t)}{\partial t}=-\frac{\beta }{{C}_{P}}H({r}^{^{\prime}})\frac{\partial I(t)}{\partial t}$$

Refer to (1), ${c}_{s}$ represents the speed of sound, ${C}_{p}$ and $\beta$ represent the specific heat capacity and volume expansion coefficient of the target body respectively, while $r$ and ${r}^{^{\prime}}$ respectively indicate the position of the ultrasound probe and the sound source. $H({r}^{^{\prime}},t)$ is the Joule heat absorbed by the biological tissue per unit time and volume, and it can be expressed as $H({r}^{^{\prime}},t)=H({r}^{^{\prime}})I(t)$, among which, $H({r}^{^{\prime}})$ indicates the spatial heat absorption distribution, and $I(t)$ is the time domain distribution of pulse excitation intensity. Using the integral solution of Green function, $p(r,t)$ can be further expressed as below in (2):

2 $$p(r,t)=\frac{1}{4\pi }\underset{\Omega }{\overset{}{\iiint }}\frac{\beta }{{C}_{p}}H({r}^{^{\prime}})\frac{\partial I(t)}{\partial t}\frac{\delta (t-|{r}^{^{\prime}}-r|/{c}_{s})}{|{r}^{^{\prime}}-r|}d{r}^{^{\prime}}$$

**Figure 1:**
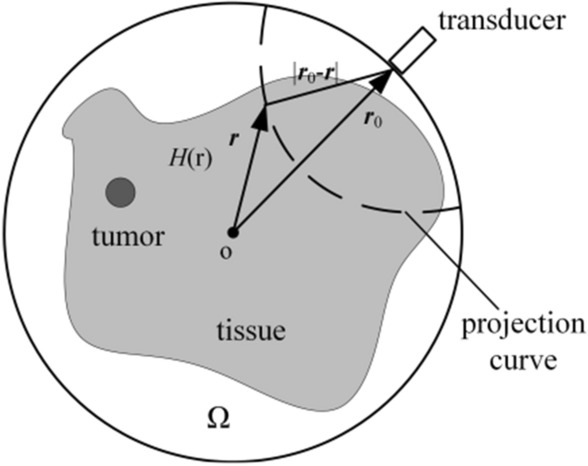
Circular scan projection principle.

Refer to (2), ${\varvec{\Omega}}$ represents the integral domain containing all sound sources, $\frac{\delta (t-|{r}^{^{\prime}}-r|/{c}_{s})}{4\pi |{r}^{^{\prime}}-r|}$ is the Green function of the sound field. According to (2), the thermoacoustic signal $p({r}_{0},t)$ detected at probe ${r}_{0}$ at time $t=|{r}_{0}-r|/{c}_{s}$ is the integral of all points along the projection arc, as shown in Fig. 1. The electrical signal ${p}_{0}({r}_{0},t)$ output by the ultrasonic probe is the result of convolution of the thermoacoustic signal $p({r}_{0},t)$ and the probe impulse response, which is expressed as below in (3):

3 $${p}_{0}({r}_{0},t)=p({r}_{0},t)*i(t)+\gamma (t)$$

Refer to (3), * represents convolution, $p({r}_{0},t)$ means an ideal thermoacoustic signal without noise, $i(t)$ is the time domain impulse response of the ultrasonic probe, and $\gamma (t)$ represents random noise. This formula can be expressed in the frequency domain as below ${p}_{0}({r}_{0},t)$:

4 $${P}_{0}({r}_{0},\omega )=P({r}_{0},\omega )I(\omega )+\gamma (\omega )$$

${P}_{0}({r}_{0},\omega )$, $P({r}_{0},\omega )$, $I(\omega )$ and $\gamma (\omega )$ are the frequency spectrum of ${p}_{0}({r}_{0},t)$, $p({r}_{0},t)$, $I(t)$ and $\gamma (t)$, respectively.

The original acoustic signal at the ultrasound probe can be restored by the Wiener filter deconvolution method, namely:

5 $$P({r}_{0},\omega )={P}_{0}({r}_{0},\omega )G(\omega )=\frac{{P}_{0}({r}_{0},\omega )I(\omega )}{|I(\omega ){|}^{2}+C}$$

6 $$p({r}_{0},t)={\mathrm{FFT}}^{-1}\left(\frac{P({r}_{0},\omega )I(\omega )}{|I(\omega ){|}^{2}+C}\right)$$

Refer to (5), $G(\omega )$ is the Fourier transform spectrum of the Wiener filter, $C=1/(\alpha |I(\omega )|)$ is the regularization factor, and $\alpha$ is a mature factor to match the ratio of the power spectrum of the input signal and the noise signal in the entire period. ${\mathrm{FFT}}^{-1}$ represents the inverse fast Fourier transform. Using formula (6), the acoustic signal ${p}_{0}({r}_{0},t)$ collected by the ultrasonic probe can be converted into the original acoustic signal $p({r}_{0},t)$.

According to the above original sound signal $p({r}_{0},t)$, the thermal sound source $H({r}^{^{\prime}})$ can be reconstructed by the Filtered backprojection algorithm, as shown below in (7):

7 $$H({r}^{^{\prime}})=-\frac{{r}_{0}^{2}}{2\pi \eta {c}_{s}^{4}}\underset{{\phi }_{0}}{\overset{}{\int }}{\left.d{\phi }_{0}\frac{1}{t}\frac{\partial p({r}_{0},t)}{\partial t}\right|}_{t=|{{r}_{0}-r}^{^{\prime}}|/{c}_{s}}$$

Refer to (7), $\eta =\beta /{C}_{p}$, and ${\phi }_{0}$ is the circumferential angle of the location where the ultrasound probe is located.

The inverse problem of acoustic field is to reconstruct the thermoacoustic source from the collected thermoacoustic signals, while the inverse problem of electromagnetic field is to reconstruct the conductivity of the measured sample from the thermoacoustic source. Assuming that the target body is an acoustically homogeneous medium with electrical conductivity of $\sigma ({r}^{^{\prime}})$, and that the ultrasonic probe and the target body are both immersed in the insulating medium, with low-frequency electromagnetic excitation and electrical quasi-static approximation, the boundary conditions and governing equations that the target area meets are described as below in (8):

8 $$\begin{gathered} \nabla \cdot {(}\sigma \nabla \varphi {)} = 0 \hfill \\ \varphi = U{(}t{) }{\varvec{r}}^{\prime} \in \sum_{1} \hfill \\ \varphi = 0 \, {\varvec{r}}^{\prime} \in \sum_{2} \hfill \\ \partial \varphi /\partial {\varvec{n}} = 0 \, {\varvec{r}}^{\prime} \in \sum_{3 - 6} \hfill \\ \end{gathered}$$

where $\phi$ represents the electric scalar potential, ${\sum }_{1}$ is the plane where the high voltage plate is located, ${\sum }_{2}$ is the ground plate plane, and ${\sum }_{3-6}$ are the other four boundaries of a cube imaging region except for the electrode. According to the governing equation given in (8), the electric field intensity in the target body can be calculated as below:

9 $$E({r}^{^{\prime}})=-\nabla \phi$$

The relationship between the thermal sound source term and the conductivity of the target body can be expressed as follows in (10):

10 $$H({r}^{^{\prime}})=\sigma |E({r}^{^{\prime}}){|}^{2}=\sigma |\nabla \phi {|}^{2}$$

Refer to (10), the scalar potential $\phi$ is also a function of the target body’s conductivity $\sigma$, therefore it is a nonlinear optimization problem to reconstruct the conductivity with known thermoacoustic source $H({r}^{^{\prime}})$.

A two-dimensional ACTAI area is established for the imaging tomographic plane, which is discretized into rectangular cells with M rows and N columns, and converted into M × N-dimensional column vectors. Let n = M × N, and assume that the conductivity inside the j-th rectangular area is uniform, where j = 1,2,…,n. According to the ACTAI forward problem model, the thermal function in the imaging area can be calculated as $H({r}^{^{\prime}})=f\in {R}^{n\times 1}$, and the thermal sound source reconstructed by filtered back projection is $y\in {R}^{n\times 1}$.

The least square method is adopted to find the optimal solution, and the optimization problem is established as follows:

11 $$\mathrm{S}=\mathrm{min}(y-f(\sigma ){)}^{T}(y-f(\sigma ))$$

Then the problem of reconstructing the conductivity of the target body $\sigma$ is transformed into finding the optimal combination of $\sigma$ to minimize the objective function $\mathrm{S}$.

The conductivity reconstruction algorithm based on least squares solves the nonlinear problem through approximate linearization. However there are problems of low image reconstruction accuracy and poor anti-noise performance. In addition, multiple iterations are often required in the calculation process, and the calculation cost is very high. Therefore, it is necessary to explore a more efficient, accurate and stable conductivity reconstruction method.

**Figure 2:**
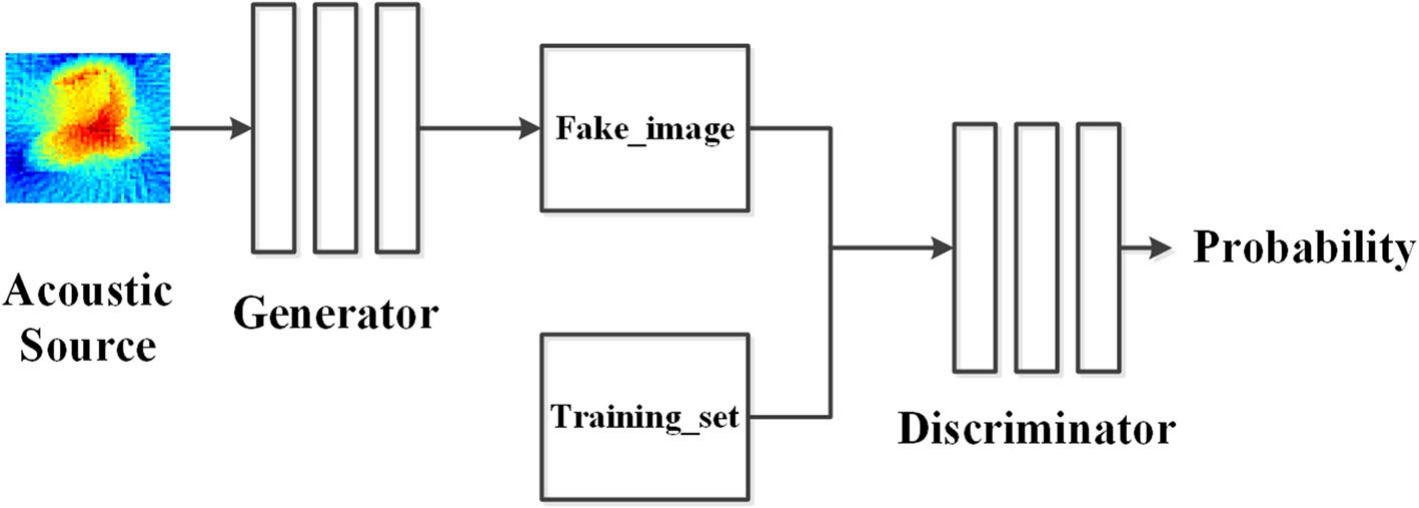
Generative adversarial network principle diagram.

The Generative Adversarial Network draws on the idea of a two-player game, and is composed of two independent units: a Generator Network (GN) and a Discriminator Network (DN). As shown in Fig. 2, in the training process, the output of GN, that is, the generated sample, is usually used as the input of DN to complete the forward propagation process of the network. For the applied current thermoacoustic imaging problem, we use the thermoacoustic source image as the input of GN, and pair the conductivity image output by GN with the real conductivity image to generate samples, which together serve as the input of DN. In the back propagation process, GN and DN networks need to be separately trained to optimize the weight and bias of each network. When training DN, the pairing of the “fake image” generated by GN and the real conductivity image is marked as FALSE; while training GN, the pairing of the “fake image” generated by GN and the real conductivity image is marked as TRUE. In other words, the ultimate goal of training the GN network is to make the “fake images” generated by the GN deceive the DN network as much as possible, so that the DN network thinks this is a real pairing. While the ultimate goal of training the DN network is to make the DN network be able to identify as much as possible the “Fake image” generated by GN network. Through batch and repeated training of a large amount of data, the probability of FALSE and TRUE in the output result of DN is each 50%, that is, the DN network cannot judge the authenticity of the image generated by the GN network. At this time, the conductivity image generated by the GN network can “mix the false with the genuine” to satisfy the matching relationship with the real conductivity image.

**Figure 3:**
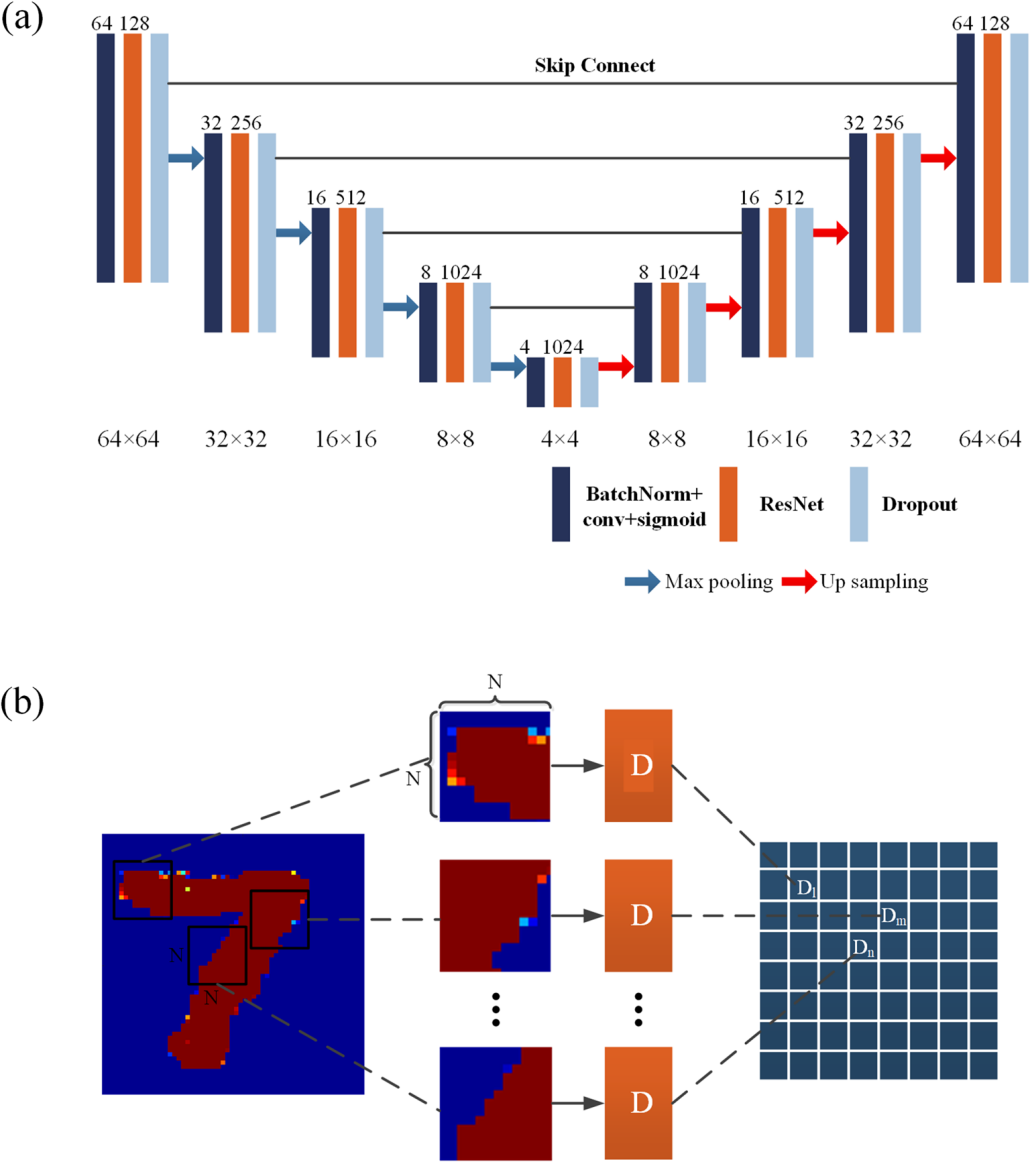
Main components of generative adversarial network (**a**) generator network; (**b**) discriminator network.

The Generator Network of GAN is demonstrated in Fig. 3a. We use an improved U-shaped convolutional neural network^20^ structure based on the residual network (ResNet). Firstly, the addition of the ResNet network avoids the degradation phenomenon that occurs with the increase in the number of network layers. Secondly, we also introduced a new convolutional layer and activation function Relu^21,22^ in the front end of ResNet^23^, and the back end uses the Dropout layer to prevent over-fitting. The U-shaped symmetric structure and jump connection mode of the generating network can further improve the problem of gradient disappearance in the training process of the deep learning network. All convolutional and deconvolutional layers used in this paper have a convolution kernel size of 3 × 3, and the sliding step size is 2. The overall structure of the number of feature maps and the size of feature maps is shown in Fig. 3a.

The Discriminator Network of GAN is demonstrated in Fig. 3b. It adopts Patch-GAN (Markov Discriminator) structure and consists of 4 fully convolutional layers. First, the image is divided into 30 N × N patches, then each patch is judged, and finally the input is mapped to a matrix X. The average value of each element in X is the final output of the discriminator.

The loss function of a standard GAN network is:

12 $${L}_{cGAN}(G,D)={E}_{x,y\sim {P}_{data}(x,y)}\left[logD(x,y)\right]+{E}_{x\sim {P}_{data}(x)}\left[log(1-D(x,G(x)))\right]$$

In addition to the loss function of standard GAN network, the loss function of pix2pix also introduces the loss function of ${L}_{1}$:

13 $${L}_{{L}_{1}}(G)={E}_{x,y\sim {P}_{data}(x,y)}\left[||y-G(x)||\right]$$

Therefore, the final GAN network's loss function is:

14 $${G}^{*}=arg\,\underset{G}{\mathrm{min}}\,\underset{D}{\mathrm{max}}\,{L}_{cGAN}(G,D)+\lambda {L}_{{L}_{1}}(G)$$

where $x$ is the input image, $y$ is the true image, $G$ is the generator, and $D$ is the discriminator. The loss of the standard GAN network is responsible for capturing the image, and ${L}_{1}$ is responsible for capturing low-frequency features such that the generated result is both true and clear.

After the GAN training, the parameters of the Generator Network are fixed and saved for the reconstruction of the conductivity image. We can then use the simulation data to test the above-mentioned GAN network. The specific process is as follows: first, for each new conductivity sample, calculate the sound field distribution in the COMSOL software according to the multi-physics coupling positive problem model, and then the measurement signal is obtained after convolution probe characteristics to solve the positive problem; secondly, the original sound signal is obtained by wiener filtering deconvolution and the sound source distribution is reconstructed through filtered back projection; finally use the reconstructed sound source as the input of the generator in the GAN network, the output of which is the conductivity image to be solved.

## Result

**Figure 4:**
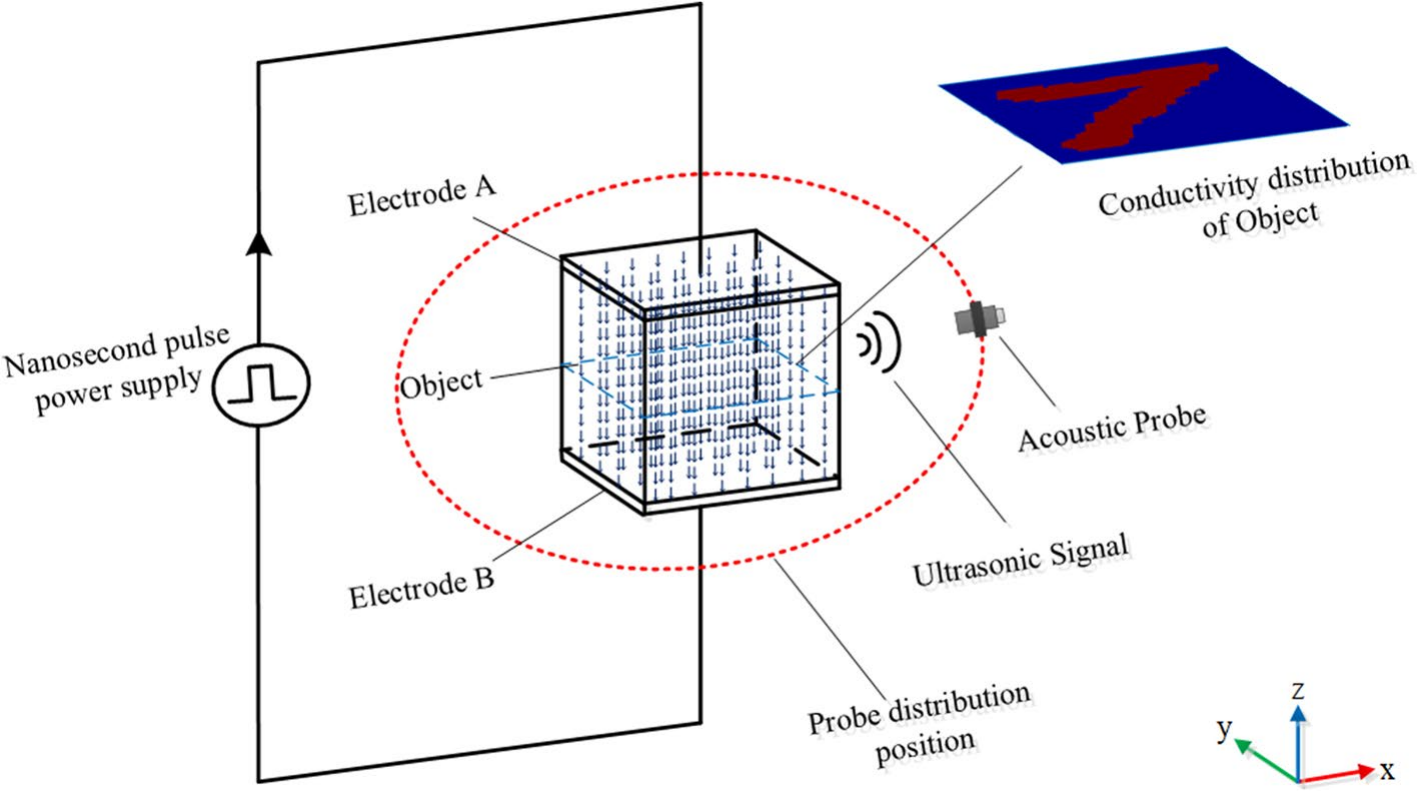
Applied current thermoacoustic imaging principle.

The simulation model shown in Fig. 4 is established, and the conductivity samples adopt the MNIST handwritten digit set that has been widely used in machine learning. The size of the target volume is $4\mathrm{cm}\times 4\mathrm{cm}\times 4\mathrm{cm}$, and its xoy interface is evenly divided into $64\times 64$ grids. The handwritten digital image is assigned to the target plane as the conductivity distribution data and its size is consistent in the Z direction, in which the digital region conductivity is set as 1 and the conductivity of other regions is set as 0. $1000$ handwritten digital images are selected as conductivity samples, and the electric field intensity, thermal sound source and sound field distribution are calculated by formulas (1), (8) and (10) using the finite element method. The 1000 sets of data obtained are randomly divided into two groups, with one set of 900 pieces targeted for network training, and the other set of 100 pieces for network testing.

**Figure 5:**
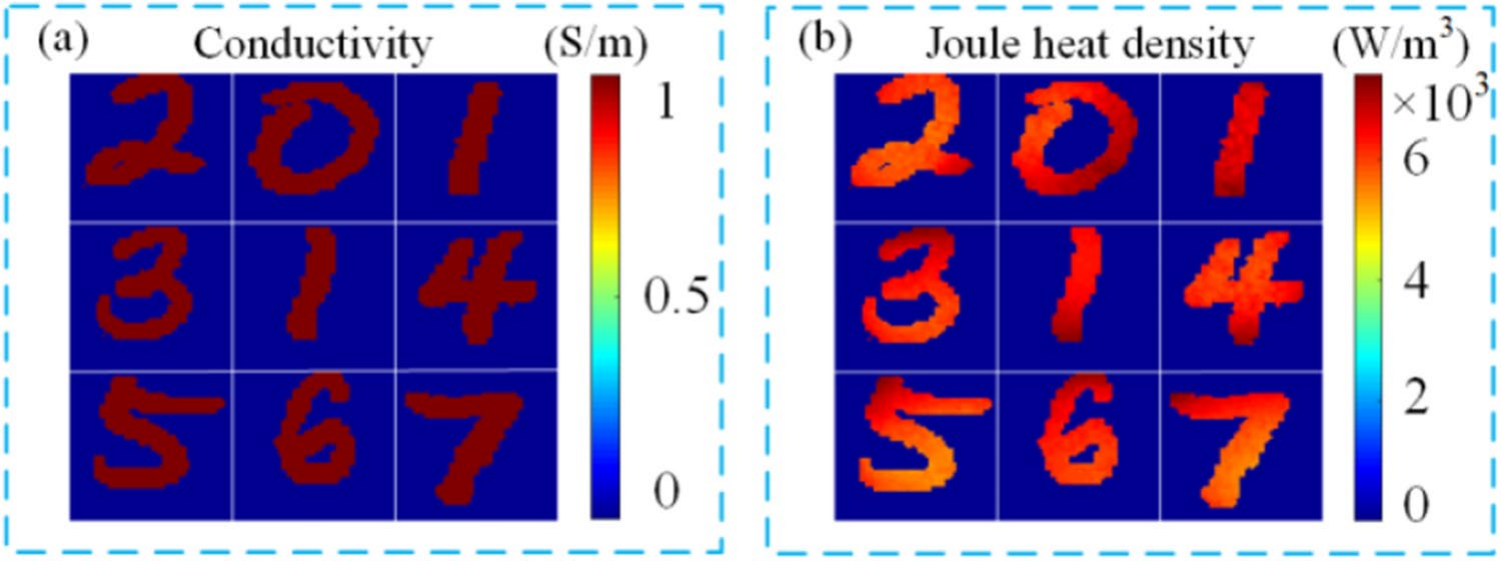
Calculation results of electric field (**a**) conductivity; (**b**) Joule heat density.

Assuming that the volume expansion coefficient of the tissue is $\beta =3.8\times {10}^{-4}{\mathrm{ K}}^{-1}$, the heat capacity is ${C}_{p}\approx 3.94 \mathrm{mJ}/(\mathrm{g mK})$, the propagation velocity of the thermoacoustic signal in the tissue and the ultrasonic coupling medium is $1404\mathrm{ m}/\mathrm{s}$, and the voltage applied by the electrode plate is $U(t)={10}^{4}\times {\mathrm{e}}^{\frac{-(t-\mathrm{b}{)}^{2}}{2{\mathrm{c}}^{2}}}$, where $\mathrm{b}=1\times {10}^{-6}$ and $\mathrm{c}=1\times {10}^{-7}$, then the thermal sound source generated by the target body at time $0.8 \mu s$ is shown in Fig. 5.

**Figure 6:**
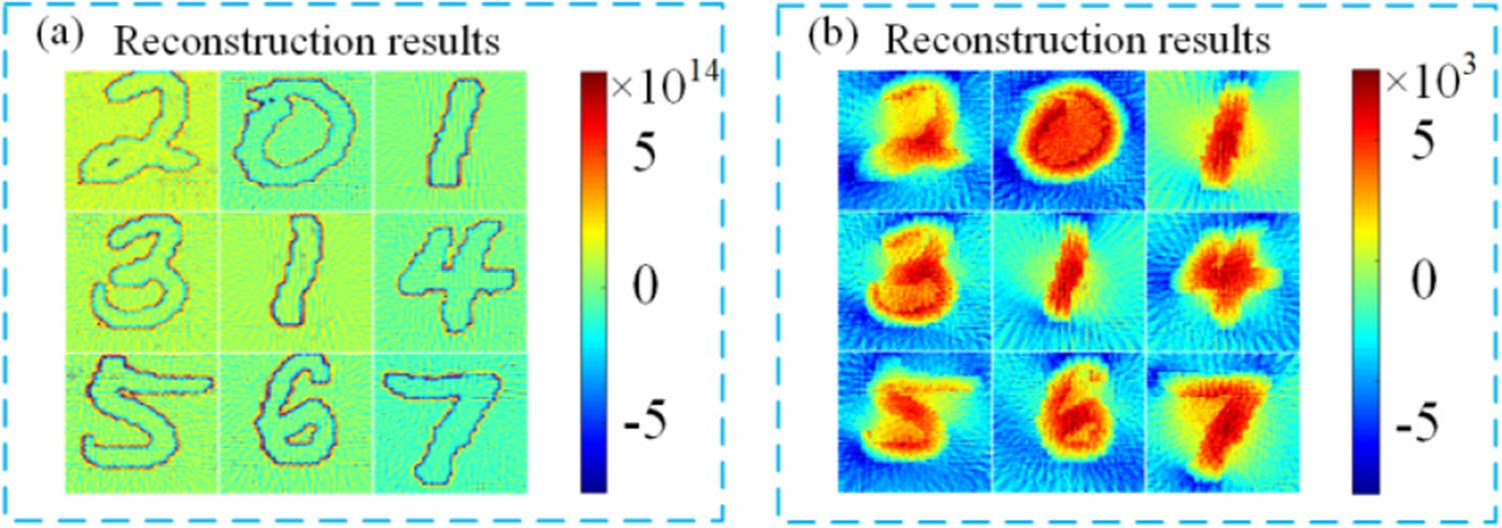
Calculation results of acoustic field: (**a**) direct reconstruction; (**b**) reconstruction after preprocessing.

If 72-channel ultrasound probes are placed around the target, then the acoustic signal they receive contains the frequency response characteristics of each probe. Figure 6 below demonstrates the comparison between the reconstructed sound source directly using FBP and the reconstructed sound source after preprocessing the sound signal with Wiener filter deconvolution. It can be found that the sound source reconstructed after deconvolution using Wiener filtering can more accurately restore the sound source distribution inside the target body. Due to the limitation of calculation amount, this paper only divides the tomographic plane into $64\times 64$ grids. For future research, a tighter grid division can be used to get higher-quality results of sound source reconstruction.

**Figure 7:**
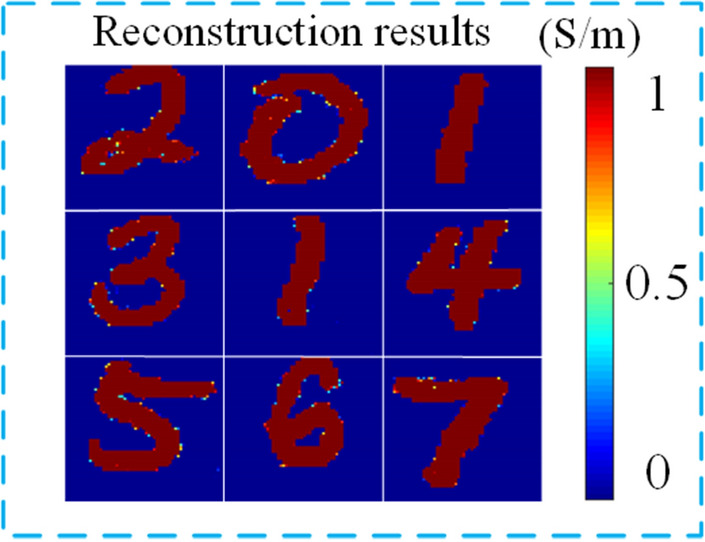
Reconstruction results of conductivity.

Input the sound source in Fig. 6b as a test sample into the GAN network, and the reconstruction result is shown in Fig. 7.

Structural Similarity (SSIM) and Peak Signal to Noise Ratio (PSNR) evaluation indicators are introduced to perform quantitative evaluation on the reconstructed image quality of conductivity, which is expressed in the following formula:

15 $$\mathrm{SSIM}(x,y)=\frac{(2{\mu }_{x}{\mu }_{y}+{c}_{1})(2{\sigma }_{xy}+{c}_{2})}{({\mu }_{x}^{2}+{\mu }_{y}^{2}+{c}_{1})({\sigma }_{x}^{2}+{\sigma }_{y}^{2}+{c}_{2})}$$

Refer to (15), ${\mu }_{x}$ is the average value of image $x$, ${\mu }_{y}$ is the average value of image $y$, ${\sigma }_{x}$ is the variance of $x$, ${\sigma }_{y}$ is the variance of $y$, and ${\sigma }_{xy}$ is the covariance of $x$ and $y$. ${c}_{1}=({k}_{1}L{)}^{2}$, ${c}_{2}=({k}_{2}L{)}^{2}$ is a constant used to maintain stability, usually taking the following values: ${k}_{1}=0.01$, ${k}_{2}=0.03$, $L=255$. The value range of SSIM is 0–1, and when two images are exactly the same, the value of SSIM is 1.

When solving the PSNR value, it is necessary to first calculate the Mean Squared Error (MSE) of each target image, as indicated in (16):

16 $$\mathrm{MSE}=\frac{1}{mn}\sum_{i=0}^{m-1}\sum_{j=0}^{n-1}||\mathrm{I}(i,j)-\mathrm{K}(i,j)|{|}^{2}$$

Then calculate the Peak Signal to Noise Ratio of the reconstructed image according to the MSE, as shown in (17):

17 $$\mathrm{PSNR}=10{\mathrm{log}}_{10}\left(\frac{{\mathrm{MAX}}_{\mathrm{\rm I}}^{2}}{\mathrm{MSE}}\right)$$

In the above formula, ${\mathrm{MAX}}_{\mathrm{\rm I}}$ is the maximum pixel value in image I. The larger the PSNR value, the smaller the distortion. The conductivity reconstruction quality in Fig. 7 is shown in Table 1. Compared with Fig. 6, GAN not only accurately reconstructs the conductivity image of the target body, but also effectively suppresses the ripple noise generated during the reconstruction process. It is also worth mentioning that a well-trained GAN network can reconstruct a conductivity image in less than 1 s, which is much more efficient than traditional algorithms.

**Table 1:** Reconstruction quality of digital conductivity samples.

Ground truths for testing	SSIM	PSNR
	0.9271	21.7999
	0.9486	23.0278
	0.9910	31.4483
	0.9529	23.1697
	0.9788	28.3142
	0.9687	24.6419
	0.9515	23.6432
	0.9494	22.8817
	0.9716	24.8629

**Figure 8:**
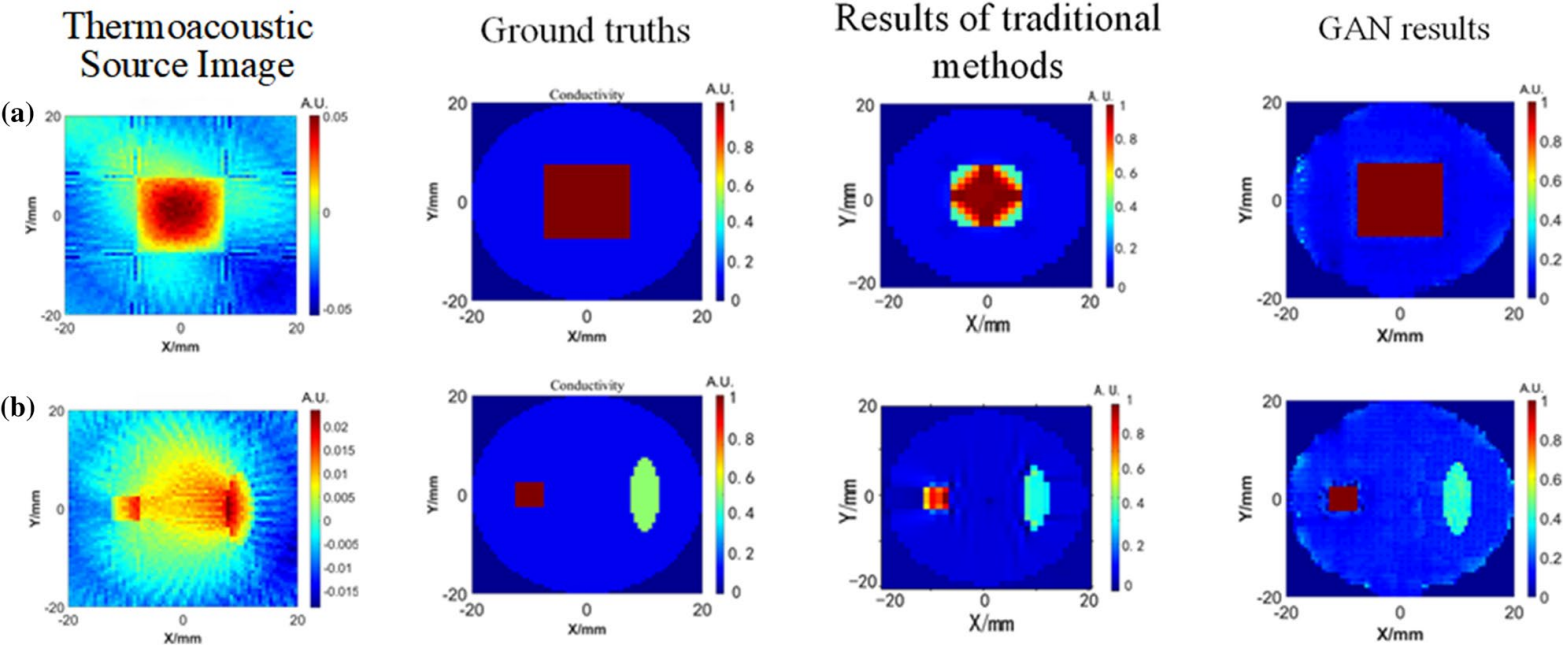
Thermoacoustic source images and conductivity reconstruction results of the models (**a**) simple model (**b**) complex model.

For the network trained by digital conductivity samples, we continue to use digital conductivity samples for testing. Although the test samples have never appeared in the training set, they are still lack of persuasion. Therefore, we use the sample which is quite different from the digital conductivity to test. In Fig. 8, the image of the thermoacoustic source as an input to the GAN network is shown. At the same time, in order to better verify the superiority of conductivity distribution reconstruction by GAN network, the conductivity reconstruction results of traditional method are also given for comparison. The traditional method used in this paper is the least squares iterative algorithm^24^. By giving an initial conductivity value, the solution of conductivity is transformed into the solution of the objective function of conductivity. On the premise of meeting the calculation accuracy, the optimal solution is the conductivity. The conductivity reconstruction quality in Fig. 8 is shown in Table 2.

**Table 2:** Reconstruction results of the test model.

Ground truths for testing	Method	SSIM	PSNR
	GAN	0.7319	23.6804
	Traditional method^24^	0.4094	11.5926
	GAN	0.7349	19.4738
	Traditional method^24^	0.4133	11.4914

It can be found that compared with the traditional method, the conductivity image reconstructed by GAN has less artifacts and higher resolution. In addition, GAN takes less time to reconstruct the conductivity image, which meets the real-time requirements of medical imaging.

**Figure 9:**
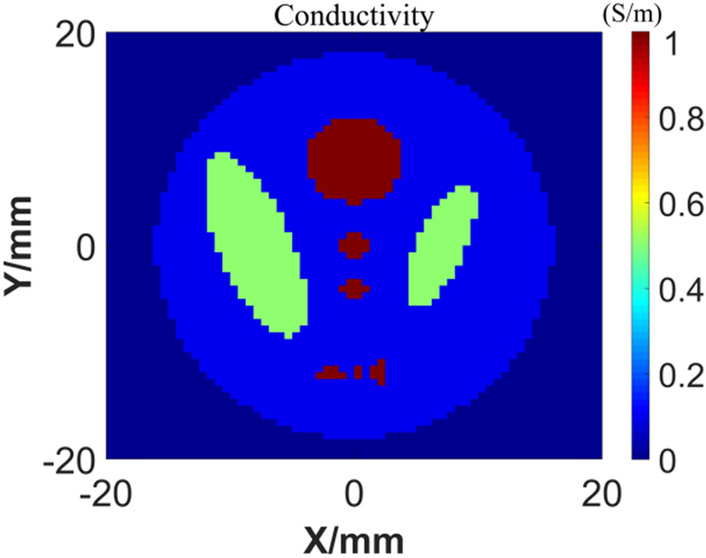
Conductivity distribution of the models.

**Figure 10:**
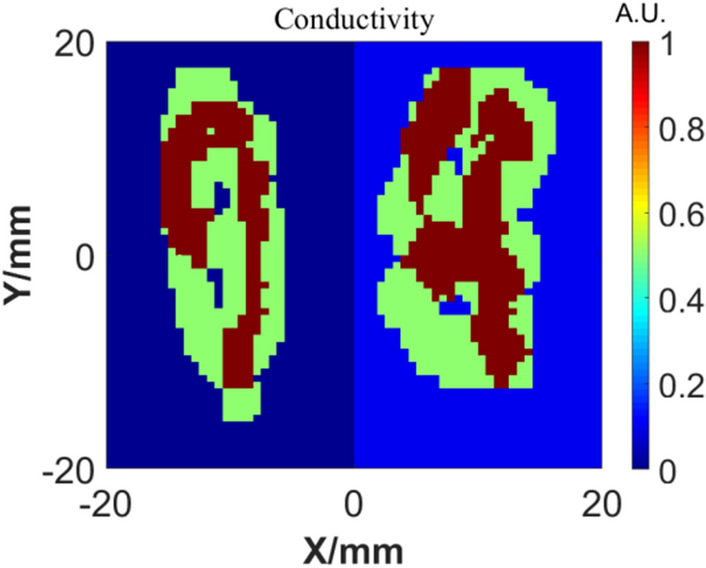
Complex training samples.

We also tested the generalization ability of the GAN network: after training the network with handwritten digital samples, other samples that are significantly different from the training samples (handwritten digital samples) are used for imaging tests. Firstly, establish the phantom model of conductivity distribution as shown in Fig. 9. Set the major axis of outer ellipsoids to be 18 mm and its conductivity to be 0.1 S/m, the conductivity of two internal centered big ellipsoids to be 0.5 S/m and conductivity of other internal ellipsoids to be 1 S/m. According to the test sample, during the GAN network training, we make a complex upgrade to the traditional MNIST data set: any two different handwritten numerals are merged to form an irregular graph, and the conductivity is inconsistent. Although the background of phantom model is not uniform, the different conductivity values can be found in the training set. The complex training samples are shown in Fig. 10.

**Figure 11:**
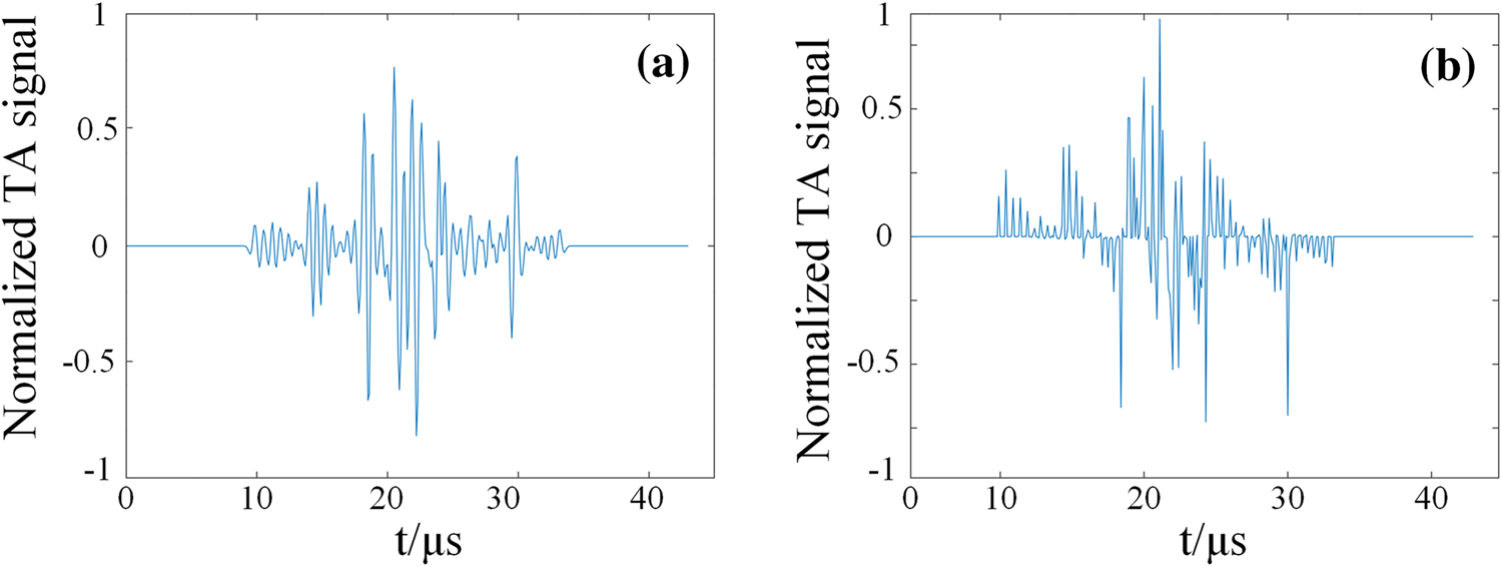
Acoustic signal (**a**) acoustic signal at ultrasonic probe (**b**) acoustic after preprocessing.

Secondly, place the ultrasound probe with a dominant frequency of 1 MHz at 0.02 m from the center of the phantom. When then probe position is at (− 0.02, 0) m, the time curve of the received acoustic signal is shown in Fig. 11a, and the time curve of acoustic signal after Wiener filter deconvolution is shown in Fig. 11b.

**Figure 12:**
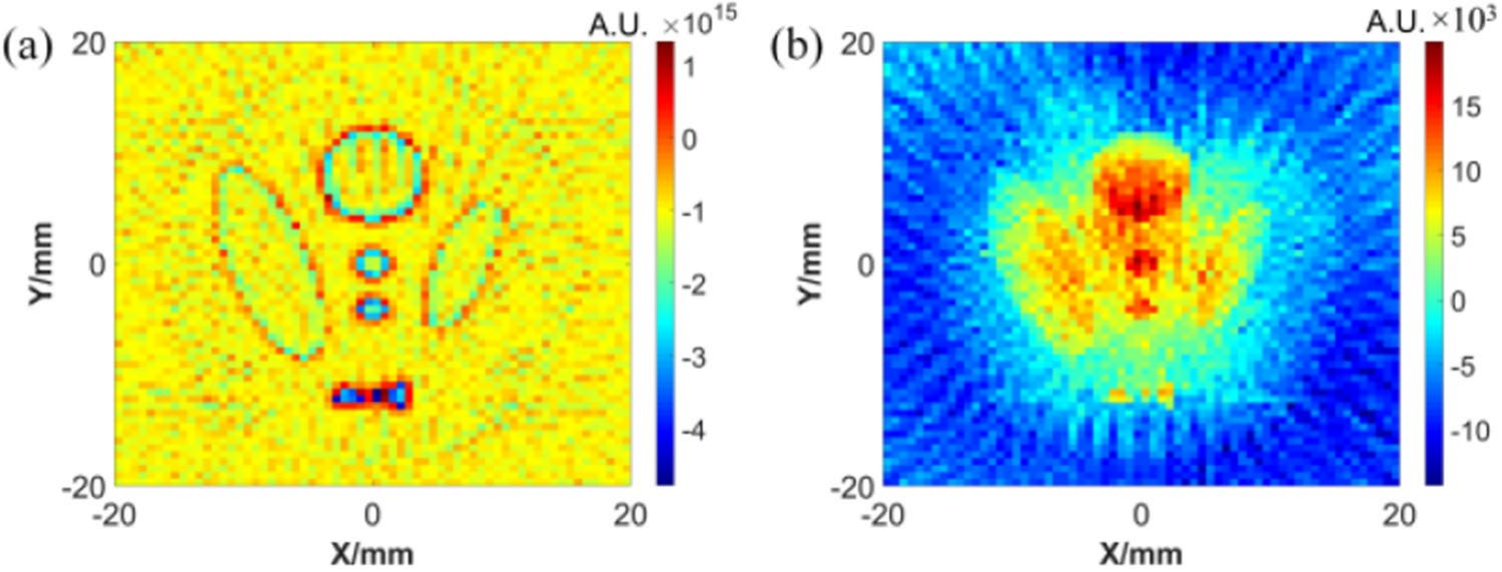
Reconstruction results of acoustic source (**a**) direct reconstruction (**b**) reconstruction after preprocessing.

Thirdly, Fig. 12 shows two results of two different reconstruction methods with the FBP algorithm: one is with the detected signal directly, and the other is with signal preprocessed by Wiener filter deconvolution.

**Figure 13:**
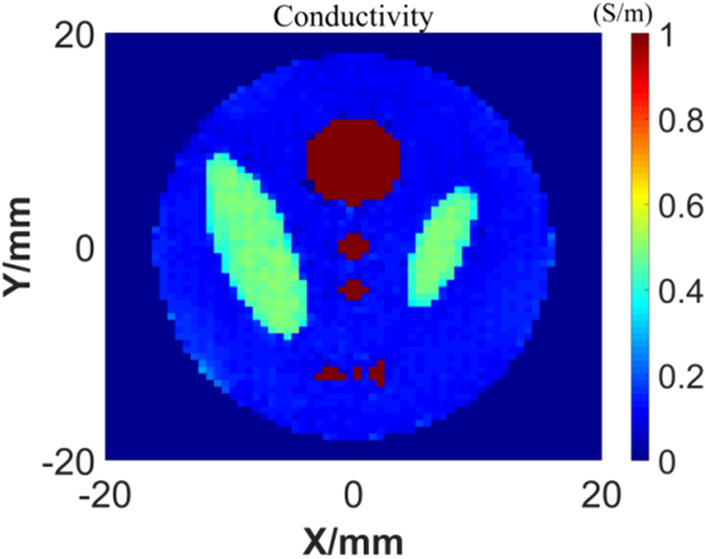
Reconstruction results of conductivity.

The next step is to take the sound source image of Fig. 12b as the input of GAN, and reconstruct the conductivity image. The results are shown in Fig. 13 and Table 3. It can be found that although some noisy points are generated at the boundary of the reconstruction result, its main distribution is basically consistent with the real model.

**Table 3:** Conductivity reconstruction quality of simulation models.

Ground truths for testing	SSIM	PSNR
Phantom model	0.9271	21.7999

**Figure 14:**
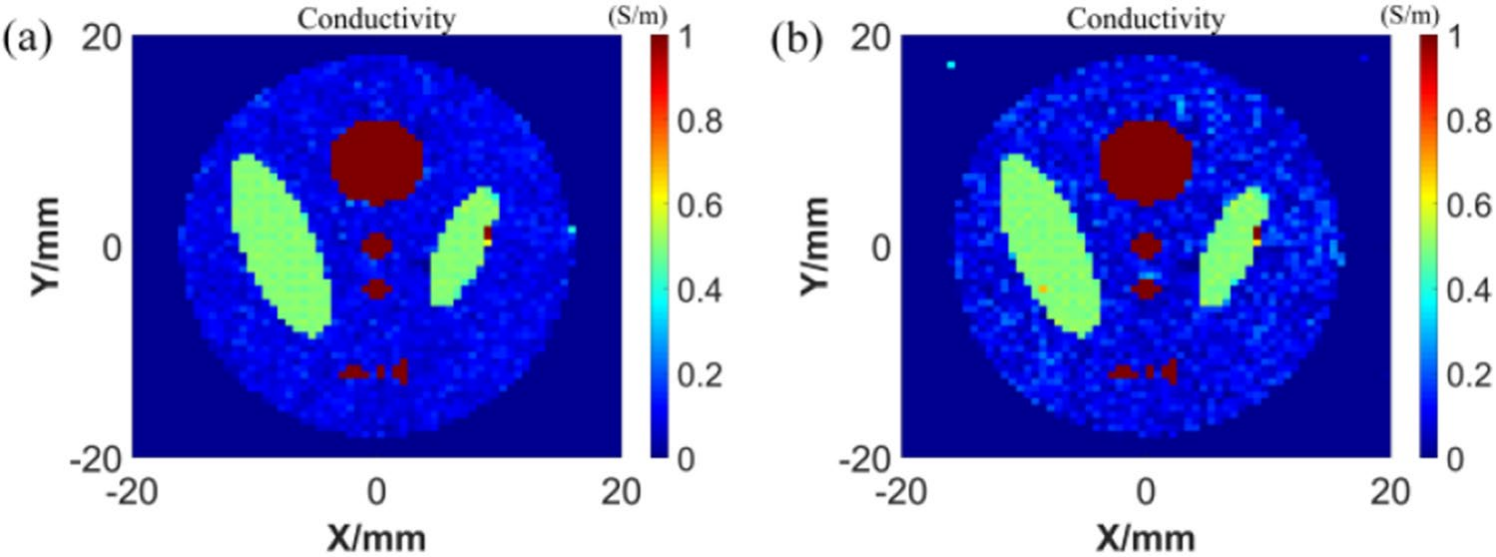
Reconstruction results of conductivity (**a**) SNR = 20 dB (**b**) SNR = 10 dB.

Finally, in order to verify the anti-noise performance of the GAN network, white noise of different intensities is introduced into the thermal sound source image. Figure 14 displays the conductivity reconstruction results after applying noise with different Signal to Noise Ratio (SNR). It can be found that the error between the reconstructed conductivity and the conductivity of the original model increases as the SNR decreases. GAN network can get better reconstruction results when the SNR is 20 dB and 10 dB.

**Figure 15:**
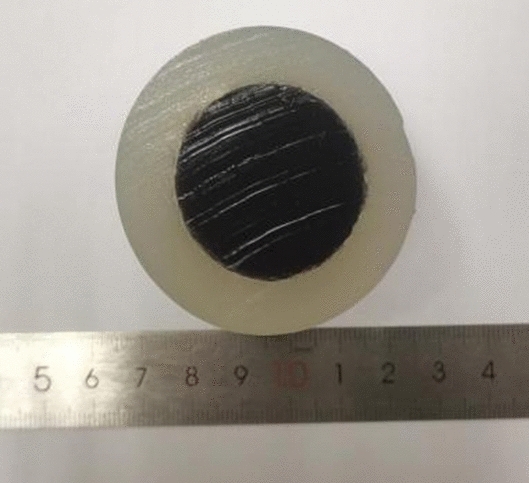
Multiconductivity gel mimics.

In order to ensure the feasibility of the method, we use cooled sodium chloride gel solution to prepare the target, and the concentration of sodium chloride determines the conductivity of the target. We conducted experiments on a mimic composed of two different electrical conductivities, and the measured model is shown in Fig. 15, in which the conductivity was for the central portion of the mimic, at the periphery of the mimic, the outer circle diameter of the mimic was about 6 cm, and the inner circle diameter of the mimic was about 3.5 cm.

**Figure 16:**
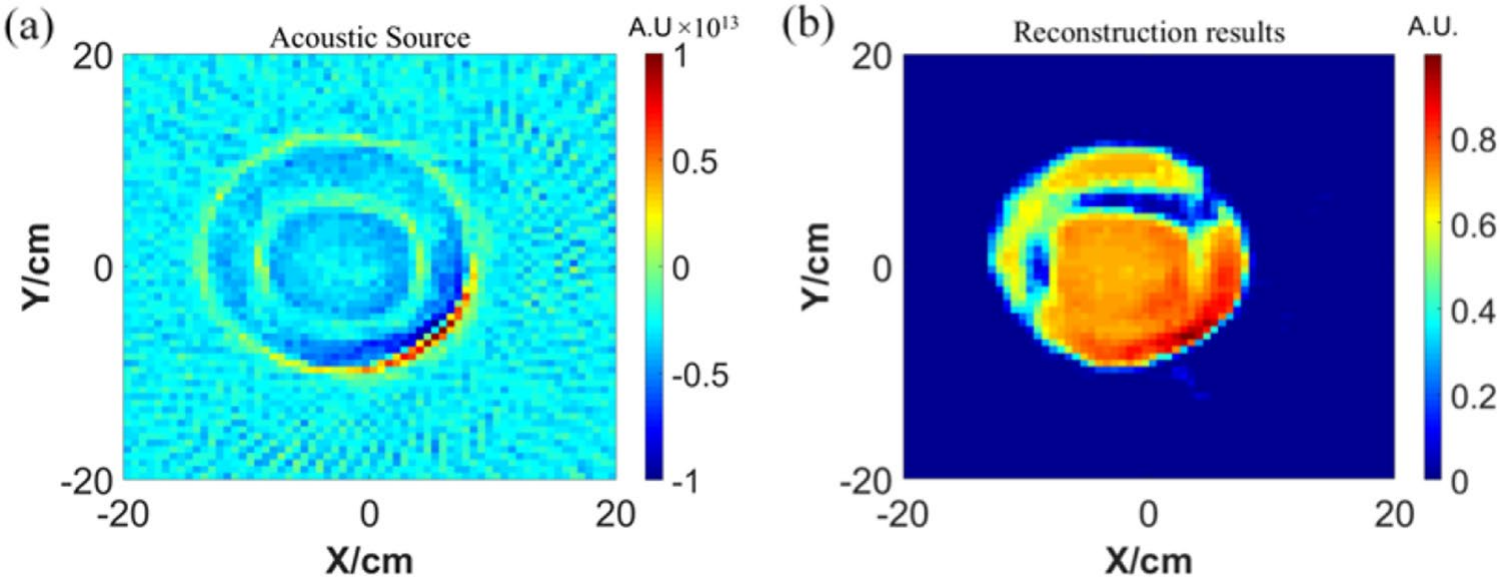
(**a**) Reconstruction of the acoustic source by the FBP (**b**) reconstructed conductivity results of GAN network.

The sources were reconstructed using filtered back projection method. The reconstruction results are shown in Fig. 16a. It can be seen that for the measured acoustic signal received by the ultrasound probe using the filtered back projection method, the conductivity information at the target body contour can be reconstructed more completely, but how its inner specific conductivity distribution can not be reconstructed well and the reconstruction results have more serious artifacts.

After obtaining the preliminary sound source reconstruction results, input them into the built GAN network for conductivity imaging. The results are shown in Fig. 16b. It can be seen that the designed network can still reconstruct the conductivity distribution of the target according to the measured ultrasonic data.

## Discussion

Although the network we built is specifically trained on MNIST handwritten digit samples, satisfactory reconstruction results can still be obtained for other types of test samples. This indicates that in the Applied Current Thermoacoustic Imaging system, the GAN network has learned the mapping relationship between the input thermoacoustic source and the output conductivity. In other words, it can not only complete the image matching between input and output, but also has the ability to learn nonlinear problems.

To conclude, this study focuses on Applied Current Thermoacoustic Imaging, mainly exploring the reconstruction method of inverse problem based on GAN network. Firstly, a physical model of electromagnetic and acoustic fields of Applied Current Thermoacoustic Imaging is established. Then the electric field in the model is calculated by finite element method, and the acoustic signal detected by the ultrasonic probe is simulated by the coupling relationship between electric field and acoustic field, and the thermoacoustic source distribution is reconstructed. In response to the problem of conductivity reconstruction, the research proposes an imaging method based on Generative Adversarial Network, and finally, with conductivity-based handwritten digit set training, it finally verifies that the network can learn the non-linear mapping relationship between the sound source and the conductivity, and thus realizes the deep learning reconstruction process of conductivity. Theoretical analysis and simulation results show that the method proposed in this paper can quickly and accurately reconstruct the conductivity image from the sound source image under the condition of low Signal to Noise Ratio, which verifies the applicability of the GAN network in the ACTAI problem.
